# The importance of falsification endpoints in observational studies of vaccination to prevent severe disease: A critique of a harm–benefit analysis of BNT162b2 vaccination of 5- to 11-year-olds

**DOI:** 10.1017/S0950268824000098

**Published:** 2024-02-16

**Authors:** Tracy B. Høeg, Alyson Haslam, Vinay Prasad

**Affiliations:** 1 Department of Epidemiology and Biostatistics, University of California, San Francisco, San Francisco, CA, USA; 2 Department of Clinical Research, University of Southern Denmark, Odense, Denmark

**Keywords:** COVID-19, epidemiology, healthy vaccinee bias, observational study, vaccine

## Abstract

We explore one systematic review and meta-analysis of both observational and randomized studies examining COVID-19 vaccines in 5- to 11-year-olds, which reported substantial benefits associated with vaccinating this age group. We discuss the limitations of the individual studies that were used to estimate vaccination benefits. The review included five observational studies that evaluated vaccine effectiveness (VE) against COVID-19 severe disease or hospitalization. All five studies failed to adequately assess differences in underlying health between vaccination groups. In terms of vaccination harms, looking only at the randomized studies, a significantly higher odds of adverse events was identified among the vaccinated compared with the unvaccinated. Observational studies are at risk of overestimating the effectiveness of vaccines against severe disease if healthy vaccinee bias is present. Falsification endpoints can provide valuable information about underlying healthy vaccinee bias. Studies that have not adequately ruled out bias due to better health among the vaccinated or more vaccinated should be viewed as unreliable for estimating the VE of COVID-19 vaccination against severe disease and mortality. Existing systematic reviews that include observational studies of the COVID-19 vaccine in children may have overstated or falsely inferred vaccine benefits due to unidentified or undisclosed healthy vaccinee bias.

## Introduction

Systematic reviews of observational studies are susceptible to the same biases as individual studies if similar unaddressed confounding variables tend to influence the results in the same direction. In this way, systematic reviews using observational data of the COVID-19 vaccines against severe disease may consistently produce results that overestate or falsely infer vaccine effectiveness (VE) if underlying healthy vaccinee bias is present in some or all of the studies. We present one potential example of this paradigm in a systematic review and meta-analysis [1] of the safety and effectiveness of messenger ribonucleic acid (mRNA) vaccination against COVID-19 in children aged 5–11. We provide examples of how the review lacked sufficient evidence to conclude that the benefits of vaccinating this demographic outweighed the harms.

### A systematic review and meta-analysis of BNT162b2 vaccination of 5- to 11-year-olds

In their review [1] of mRNA vaccine BNT162b2 efficacy in 5- to 11-year-olds, Watanabe et al. included fifteen studies, of which twelve (shown in Table 1) assessed VE. Ten of these were observational and two were randomized. However, neither of the two randomized studies evaluated the efficacy of mRNA vaccination in preventing severe disease or hospitalization as they were underpowered for these endpoints. Only one case of severe COVID-19 occurred in both studies combined.

**Table 1.** Characteristics of studies evaluating COVID-19 vaccination efficacy or effectiveness among children 5–11 years of age

Effectiveness against severe disease assessed	Author, journal, year	Study design	Population	Endpoints/confounding variables
No	Cohen-Stavi, NEJM, 2022	Retrospective cohort	Clalit Health Services, the largest integrated healthcare service provider in Israel	Did not include relevant endpoints of hospitalization or severe disease
No	Fleming-Dutra, JAMA, 2022	Case–control, test negative	Increasing Community Access to Testing (ICATT) platform was used. ICATT is an HHS programme that contracts with four commercial pharmacy chains to facilitate drive-through SARS-CoV-2 testing nationally	Did not include relevant endpoints of hospitalization or severe disease
Yes	Price, NEJM, 2022	Case–control, test negative	CDC-funded Overcoming COVID-19 Network	Provided VE adjusted for numerous potential confounders but did not provide data on the prior year’s non-COVID-19 hospitalization rates by vaccination status. The latter appear to have been available and could have been compared with unadjusted VE to estimate the magnitude of bias due to differences in underlying health
No	Fowlkes, MMWR, 2022	Prospective cohort	Prospective cohort study monitoring SARS-CoV-2 infections among participants aged 6 months to 17 years in jurisdictions in four states (Arizona, Florida, Texas, and Utah)	Did not include relevant endpoints of hospitalization or severe disease
Yes	Tan, NEJM, 2022	Prospective cohort	Administrative records from the Ministry of Health, Singapore	Provided VE adjusted for housing status and ethnic group. Did not provide data on non-COVID-19 hospitalization or mortality rates to assess for residual bias due to underlying health differences.
Yes	Sacco, Lancet, 2022	Retrospective cohort	Italian national medical record database	Only controlled for two individual characteristics (age and sex) and only three geographical variables (municipal vaccination coverage among 30- to 50-year-olds, urbanization of municipality, and weekly regional incidence of COVID-19) Did not consider differences in underlying health and vaccination status.
Yes	Klein, MMWR, 2022	Case–control	VISION Network (emergency department and urgent care encounters and hospitalizations)	There is a slight imbalance with more underlying conditions among the vaccinated children, and it is uncertain whether healthy vaccinee bias has been ruled out. This is partly because children of higher socioeconomic status may be more likely to seek medical care and have documented underlying diagnoses. The study did not provide falsification endpoints to assess for underlying bias due to health differences.
No	Creech, NEJM, 2022 mRNA-1273	RCT	79 sites in the United States and 8 sites in Canada	Did not include relevant endpoints of hospitalization or severe disease
No	Walter, NEJM, 2022 BNT-62b2	RCT	Not specified	Did not include relevant falsification endpoints of non-COVID-19 hospitalization or severe disease rates by vaccination status
MIS-C only	Zambrano, MMWR, 2022	Case–control	Centers for Disease Control and Prevention (CDC)-funded Overcoming COVID-19 (OC-19) paediatric vaccine effectiveness network	Only endpoint was MIS-C, which is not currently a relevant risk. Furthermore, the study did not provide vaccination status by race. Black and Hispanic children had historically higher rates of MIS-C and also lower rates of vaccination. This was likely to create confounding that would overstate VE. Also, the test negative design study was not able to rule out additional bias created by differences in underlying health/MIS-C propensity by vaccination status.
No	Amir, Lancet Infect Dis, 2023	Retrospective cohort	Israeli Ministry of Health database	Did not include relevant endpoints of hospitalizations or severe disease
Yes	Shi, MMWR, 2022	Case–control	COVID-NET conducts population-based surveillance for laboratory-confirmed COVID-19-associated hospitalizations	Found twice the rate of COVID-19 hospitalization for unvaccinated as vaccinated but did not provide falsification data to assess healthy vaccinee bias.

**References**

1. Cohen-Stavi CJ, Magen O, Barda N, et al. BNT162b2 Vaccine Effectiveness against Omicron in Children 5 to 11 Years of Age. N Engl J Med. 2022 Jul 21;387(3):227–236.

2. Fleming-Dutra KE, Britton A, Shang N, et al. Association of Prior BNT162b2 COVID-19 Vaccination with Symptomatic SARS-CoV-2 Infection in Children and Adolescents during Omicron Predominance. JAMA. 2022 Jun 14;327(22):2210–2219.

3. Price AM, Olson SM, Newhams MM, et al.; Overcoming Covid-19 Investigators. BNT162b2 Protection against the Omicron Variant in Children and Adolescents. N Engl J Med. 2022 May 19;386(20):1899–1909. doi:10.1056/NEJMoa2202826. Epub 2022 Mar 30. PMID: 35353976; PMCID: PMC9006785.

4. Fowlkes AL, Yoon SK, Lutrick K, et al. Effectiveness of 2-Dose BNT162b2 (Pfizer BioNTech) mRNA Vaccine in Preventing SARS-CoV-2 Infection among Children Aged 5–11 Years and Adolescents Aged 12–15 Years – PROTECT Cohort, July 2021–February 2022. MMWR Morb Mortal Wkly Rep. 2022 Mar 18;71(11):422–428.

5. Tan SHX, Cook AR, Heng D, Ong B, Lye DC, Tan KB. Effectiveness of BNT162b2 Vaccine against Omicron in Children 5 to 11 Years of Age. N Engl J Med. 2022 Aug 11;387(6):525–532. doi:10.1056/NEJMoa2203209. Epub 2022 Jul 20. PMID: 35857701; PMCID: PMC9342421.

6. Sacco C, Del Manso M, Mateo-Urdiales A, et al. Italian National COVID-19 Integrated Surveillance System and the Italian COVID-19 Vaccines Registry. Effectiveness of BNT162b2 Vaccine against SARS-CoV-2 Infection and Severe COVID-19 in Children Aged 5–11 Years in Italy: A Retrospective Analysis of January–April, 2022. Lancet. 2022 Jul 9;400(10346):97–103.

7. Klein NP, Stockwell MS, Demarco M, et al. Effectiveness of COVID-19 Pfizer-BioNTech BNT162b2 mRNA Vaccination in Preventing COVID-19-Associated Emergency Department and Urgent Care Encounters and Hospitalizations among Nonimmunocompromised Children and Adolescents Aged 5–17 Years – VISION Network, 10 States, April 2021–January 2022. MMWR Morb Mortal Wkly Rep. 2022 Mar 4;71(9):352–358.

8. Creech CB, Anderson E, Berthaud V, et al.; KidCOVE Study Group. Evaluation of mRNA-1,273 Covid-19 Vaccine in Children 6 to 11 Years of Age. N Engl J Med. 2022 May 26;386(21):2011–2023.

9. Walter EB, Talaat KR, Sabharwal C, et al. C4591007 Clinical Trial Group. Evaluation of the BNT162b2 Covid-19 Vaccine in Children 5 to 11 Years of Age. N Engl J Med. 2022 Jan 6;386(1):35–46.

10. Zambrano LD, Newhams MM, Olson SM, et al. Overcoming COVID-19 Investigators. Effectiveness of BNT162b2 (Pfizer-BioNTech) mRNA Vaccination against Multisystem Inflammatory Syndrome in Children Among Persons Aged 12–18 Years – United States, July–December 2021. MMWR Morb Mortal Wkly Rep. 2022 Jan 14;71(2):52–58.

11. Amir O, Goldberg Y, Mandel M, Bar-On YM, Bodenheimer O, Freedman L, Ash N, Alroy-Preis S, Huppert A, Milo R. Initial Protection against SARS-CoV-2 Omicron Lineage Infection in Children and Adolescents by BNT162b2 in Israel: An Observational Study. Lancet Infect Dis. 2023 Jan;23(1):67–73.

12. Shi DS, Whitaker M, Marks KJ, et al.; COVID-NET Surveillance Team. Hospitalizations of Children Aged 5–11 Years with Laboratory-Confirmed COVID-19 – COVID-NET, 14 States, March 2020–February 2022. MMWR Morb Mortal Wkly Rep. 2022 Apr 22;71(16):574–581. doi:10.15585/mmwr.mm7116e1. PMID: 35446827; PMCID: PMC9042359.

In terms of vaccination harms, looking only at the included randomized studies in Watanabe and colleagues’ review, a significantly higher odds (OR, 1.92; 95% confidence interval (CI), 1.26–2.91) of adverse events was identified among the vaccinated compared with the unvaccinated.

The review included observational studies that evaluated VE against multisystem inflammatory syndrome in children (MIS-C), but this condition has essentially disappeared as of March 2022 [2]. Furthermore, this study was not able to rule out that the association between vaccination and lower rates of MIS-C was simply a result of subgroups that were higher risk for this condition and were less likely to be vaccinated (Table 1). Finally, any effect of vaccination against infection has been found to be marginal and does not last beyond 2–3 months [9], and the value of slightly delaying infection is questionable for most children.

The five remaining observational studies that evaluated effectiveness against severe disease or hospitalization due to COVID-19 (Table 1; the five highlighted in grey) failed to adequately assess bias created by differences in health between vaccination groups. No falsification endpoints were provided, which would have provided the most direct way to assess underlying healthy vaccinee bias.

### Healthy vaccinee bias and the utility of falsification endpoints

Historically, influenza VE studies have been prone to bias due to differences in underlying health, and most typically report better health among individuals who are vaccinated compared with those who are unvaccinated [3–5]. This bias is often referred to as ‘healthy vaccinee bias’ [3, 4]. Post hoc adjustments for differences between the groups have been found to reduce this bias but may still be insufficient [6]. Specifically, adjusting for differences in rates of underlying diagnoses may be misleading if healthier people of higher socioeconomic status are more likely to seek medical care and receive official diagnoses.

Although there is an abundance of observational data suggesting the benefit of influenza vaccines against hospitalization and death, pooled randomized data have to date failed to find this benefit at any age [7–9], potentially because of the elimination of underlying healthy vaccinee bias. COVID-19 hospitalization risk, similar to influenza risk, is dependent on underlying health [10].

Observational studies conducted in many countries, including Israel [11], Austria [12], the Czech Republic [13] and the United States [14], of mRNA vaccines for severe disease with COVID-19 appear, at least in certain populations, to face the same challenge of better underlying health in those who are vaccinated against COVID-19 compared with individuals who are unvaccinated [14]. In the United States, unvaccinated adults in one healthcare organization have been found to have a 70% higher rate of all-cause mortality than those fully vaccinated with mRNA vaccines [14]. This suggests a large amount of healthy vaccinee bias in the United States as reducing COVID-19 mortality alone would not reduce overall mortality by anywhere near 70%. Some degree of healthy vaccinee bias is also suspected in children, based on uptake data of the measles, mumps, and rubella (MMR) vaccine [15]. Also, like adults, children who get vaccinated are different from those who do not. In the United States, unvaccinated children are more likely to come from households with income below the poverty level, have parents with a lower education level, be uninsured, and Black [16]. Specifically, unvaccinated 5- to 11-year-old children in the United States during the delta and omicron variants were more likely to be Black and have a higher social vulnerability index scores (the latter determined using numerous socioeconomic factors) [17].

Falsification endpoints [18], a type of negative control [19], are outcomes that can be used to detect confounding by being minimally or unaffected by the exposure of interest (in this case vaccination) but likely to reflect an intrinsic quality that affects the outcome of interest (in this case the risk of severe COVID-19). Off season and, preferably, preseason mortality rates stratified by vaccination group have been suggested as falsification endpoints [3, 4] and used in influenza vaccine studies to detect residual healthy vaccinee bias [6].

Falsification endpoints for detecting bias in studies examining VE against severe disease, hospitalization, or death from COVID-19 may include all-cause or non-COVID-19 hospitalization, intensive care unit admission, or mortality rates. These are useful falsification endpoints because they provide real-time windows into current health status. Other potential confounding variables, such as underlying diagnoses, are less useful in that they may be higher in those who seek medical care more often or may be lagging or outdated. Other confounding variables, such as race or socioeconomic status, may at best only provide partial or limited data on underlying health. Thus, though many studies provide information on potential confounding variables, it should not be assumed that the entirety of the underlying bias has been identified and addressed.

For example, two recent COVID-19 VE studies provided falsification data that added valuable context. The first is a study [12] of 3 million previously-infected Austrians of all ages, which did provide falsification data, indicating a 29% lower all-cause mortality among those with four vs. three vaccine doses, with no significant reduction in COVID-19 mortality (relative VE: −24% (95% CI, −120 to 30)) associated with the fourth dose of vaccine compared with the third. In the second study from Israel [11], those ≥50 years had an initially undisclosed 95% lower non-COVID-19 mortality rate among those with a first booster dose vs. those who had only had the primary series, which may have accounted for the entire COVID-19 mortality benefit that had been attributed to the booster, but at the very least suggested a large amount of underlying bias.

### Spotlight on individual studies

One study by Klein et al. [20] (Table 1), included in the systematic review of the COVID-19 vaccine in children 5–11 years, tested for SARS-CoV-2 in the emergency department and reported slightly higher rates of chronic conditions among the vaccinated but substantially higher rates of unvaccinated status among racial minorities, who may have been less likely to seek care, thus decreasing their opportunities to have underlying conditions diagnosed. Non-COVID-19 hospitalization and intensive care admission rates by vaccination status would have helped quantify differences in underlying health by vaccination status.

A study by Price et al. [21], included in the review (Table 1) reported an adjusted VE of 68% (95% CI, 42–82) against COVID-19 hospitalization among 5- to 11-year-olds who received the BNT162b2 primary series. It was, however, unclear whether their adjustment was sufficient to eliminate confounding by differences in underlying health. The Israeli study mentioned above [11, 22] reported an adjusted VE of 90% against hospitalization with the first booster, and the non-COVID-19 mortality rate among the unboosted was 95% higher [11], which was the same as their unadjusted VE [11], demonstrating how adjusting, even for numerous health and socioeconomic factors, can be far from adequate in terms of eliminating bias. Notably, the study by Price et al. provided information on all-cause hospitalizations in the previous year but not by vaccination status. Had the data been stratified by vaccination status, they could have more fully assessed the magnitude of bias due to differences in underlying health.

A third noteworthy observational study [23], not included in the Watanabe et al. review, found a 39% reduction in severe disease following receipt of the primary series in 5- to 11-year-old children. The authors did attempt to provide falsification information by looking at differences in VE against infection from days 4 to 10 but apparently lacked power to perform this analysis for severe disease. Data on non-COVID-19 hospitalization and mortality rates by vaccine groups would have provided helpful information in this case. Additionally, with the effectiveness against infection and severe disease being essentially the same in this study, it was unclear whether the observed decreased rate of severe disease, if not attributable to differences in underlying health, may have alone been due to postponing the infection risk window.

### Alternative methods to assess for healthy vaccinee bias

Randomized trials, by design, minimize the risk of healthy vaccinee bias. This is not inherent to most observational data sets. When using observational data to assess VE, there are additional methods that can help minimize or identify healthy vaccinee bias. One is the use of regression discontinuity design, which follows vaccinated and unvaccinated cohorts’ COVID-19 and non-COVID-19 hospitalization or mortality rates over time, before and after a discrete vaccination rollout period and determines whether there is a corresponding discontinuity (or decrease in only COVID-19 hospitalization or death) specific to the vaccinated [24].

Alternatively, one can identify evidence of healthy vaccinee bias if a decrease in death rate among the vaccinated occurs too quickly to be attributable to the vaccine [25]. This bias can be demonstrated visually in a Kaplan–Meier curve, which reveals a difference in death rates by vaccination status within days of the first dose of vaccination (before an effect against death is expected).

### Uncertain benefit of childhood COVID-19 vaccination against meaningful health outcomes

In the absence of demonstrably non-confounded analyses of relevant endpoints, we argue the benefit of mRNA vaccination against severe disease, hospitalization, or death in 5- to 11-year-olds remained unclear. Meanwhile, adverse event rates were significantly higher among the vaccinated, as demonstrated in the included randomized studies. To our knowledge, a net benefit of vaccinating this demographic using demonstrably unconfounded data has not to date been demonstrated.

## Conclusion

Observational studies of COVID-19 VE must be suspected of being confounded by healthy vaccinee bias if falsification data are not provided. Post hoc adjustments may be wholly inadequate for controlling for underlying bias [11]. Specific natural experiment study designs, such as regression discontinuity, may allow for causal inference if falsification data are included [25]. Adequately powered randomized studies remain an even more reliable way to determine VE against severe disease.

The burden lies with the researchers publishing observational studies to demonstrate that healthy vaccinee bias has not affected their VE results. Studies that have not demonstrated this cannot reliably be used in systematic reviews, risk–benefit analyses, or population-wide vaccination guidelines.

## Data Availability

Our findings are based on the published scientific articles cited in the text and do not rely on our own data set or code.
